# Perceived union support, work meaningfulness, and well-being: evidence from an emerging country context

**DOI:** 10.3389/fpsyg.2026.1839771

**Published:** 2026-08-20

**Authors:** Deniz Dirik, Ergün Metin

**Affiliations:** Department of Business Administration at Manisa Celal Bayar University, Manisa Celal Bayar University, Manisa, Türkiye

**Keywords:** job satisfaction, perceived union support, status anxiety, union membership, work meaningfulness

## Abstract

**Introduction:**

Meaningful work is a key determinant of employee well-being, yet little is known about the role of collective institutions such as trade unions in shaping perceptions of work meaningfulness. Drawing on meaningful-work literature, this study examines whether perceived union support (PUS) relates to employees’ work meaningfulness and, through it, to job and life satisfaction.

**Methods:**

Survey data were collected from 1,673 public employees in Türkiye. Regression-based mediation and moderated mediation analyses were conducted using open access Jamovi software.

**Results:**

According to findings, PUS is associated with higher levels of meaningfulness beyond membership status and demographics. Meaningfulness, in turn, is positively associated with both job satisfaction and life satisfaction, with small but reliable indirect effects from PUS to job and life satisfaction. Union membership, per se, was not associated with meaningfulness or satisfaction. Moderation tests showed that status anxiety and job stress were each related to lower meaningfulness, yet neither altered the positive PUS-meaningfulness link.

**Discussion:**

Results corroborate the importance of the quality of workers’ union experiences, feeling supported over formal membership status for understanding meaningful work and well-being. The study contributes to the meaningful work literature by highlighting the role of collective institutions in shaping work experiences and suggest that relational engagement is critical for unions seeking to promote worker well-being.

## 1 Introduction

The concept of meaningful work, closely related to decent work, has become a fundamental component of empowerment literature and a critical psychological state at the individual level. It is considered a key motivator in identity construction and an essential element for institutional sustainability, even in today’s “enriched” or high-income jobs, where its absence remains an issue (Lepisto and Pratt, 2016). Although defining what “meaningful work” entails may seem relatively straightforward through anecdotal examples, reconciling its conceptual and theoretical diversity is challenging. The common thread across various definitions is the positive organizational and individual outcomes associated with meaningful work. For example, individuals with high perceptions of meaningful work have been found to exhibit higher levels of work commitment, job satisfaction, motivation, performance, subjective well-being, and life satisfaction (Both-Nwabuwe et al., 2017; Martela et al., 2021). However, a contemporary and nuanced understanding of meaningful work experiences in the context of public organizations, with relatively higher goal complexity and ambiguity regarding one’s contribution to society remains to be explored with quantitative evidence from different cultural contexts (Tummers and Knies, 2013).

Perceived work meaningfulness, generally defined as individuals’ subjective experience of value and significance associated with their work, is a significant aspect of work life quality as well as occupational health psychology (Martela et al., 2021) and individuals with high levels of perceived work meaningfulness are generally happier at work and in life (Allan et al., 2019). A self-determination theory perspective (Ryan and Deci, 2017) which argues that humans have three basic psychological needs, that is need for autonomy, competence, and relatedness, to perceived work meaningfulness has been used to understand how perceived work meaningfulness operates. According to this perspective, satisfaction of the basic psychological needs for autonomy, competence and relatedness contributes to a sense of meaningfulness with one’s work and the lack thereof to vice versa. When individuals are in control of their actions and surroundings (autonomy), feel competent enough to actualize their goals (competence), and are part of a community with quality connections (relatedness), their subsequent sense of work meaningfulness will be higher. This is a seemingly paradoxical claim such that individuals’ sense work of meaningfulness is not only a result of their self-oriented need satisfaction as in the case of autonomy, but also a result of their other-oriented need fulfilment as in the case of relatedness (Bailey et al., 2019; Martela and Pessi, 2018; Martela and Riekki, 2018). That is, individuals’ sense of work meaningfulness features both intrinsic and extrinsic motivators working simultaneously. Although previous research has underlined the role of “autonomy” particularly for perceptions of work meaningfulness (Martela et al., 2021), the full implications of this finding for different contexts, cultures and on different samples has not been fully addressed, including public sector organization employees in emerging countries with and without union membership.

A social exchange theory perspective (Blau, 1964; Gouldner, 1960) has long suggested that a positive treatment of employees in terms of fairness, pay, equality, relationships etc. lead them to positively reciprocate, and support their organization by nurturing higher levels of positive employee attitudes such as commitment (or at least obligates them to inflict no harm to their organization; Eisenberger et al., 2001). Perceived organizational support determines the degree to which employees feel their contributions to be valued, and its absence leads to lower levels of identification, and even results in disidentification (Zagenczyk et al., 2011). Recent research has also shown that perceptions of organizational support extend beyond one’s employing organization and includes other organizational memberships that address their multifaceted problems such as trade unions (Cardador et al., 2019).

In this study, we address the potential relationship between union membership and perceived union support and work meaningfulness, and the roles of other wellbeing variables at the intersection of labor relations and organizational behavior on a large sample representative of public employees in an emerging country context. Previous literature has criticized the study of work meaningfulness in a siloed manner with simplistic designs and relying on few variables (Rosso et al., 2010). We address a neglected question in the meaningful-work literature: whether employees’ perceptions of union support, above and beyond formal union membership, explain variance in meaningfulness and downstream well-being. We test a mediation model in which perceived union support relates to job and life satisfaction via meaningfulness, and probe whether status anxiety and job stress condition this association. This approach clarifies whether the subjective quality of the union–member relationship, rather than membership per se, is the more proximal correlate of meaningful work and well-being. We operationalize several sources likely to interact with each other to significantly shape and influence public organization employees’ work meaningfulness perceptions. Given the significant role of organizational memberships, including unionization in shaping meaningfulness (Cardador et al., 2019), we expect union support to positively influence work perceptions and be consequential in terms of positive work outcomes.

## 2 Work meaningfulness

Despite contentions about its conceptualization, meaningfulness is generally accepted to be a multidimensional construct, and work meaningfulness refers to a positive subjective experience, incorporating both the self (in the form of self-actualization) and others (in the form of serving others’ needs; Lepisto and Pratt, 2016; Lips-Wiersma and Wright, 2012). Meaningfulness has epistemologically been treated as a positive construct, in direct opposition to alienation (Tummers and Knies, 2013). Bailey et al.’s (2017) definition of meaningful work as “*work that is personally enriching and that makes a positive contribution”* captures the bipartite understanding of meaningfulness, one that balances self fulfilment with prosocial impact. Everyone attaches a different “meaning” to their work, be it a means of making ends meet, status, game, career, calling, etc. (Duffy and Dik, 2013). However, meaningfulness of one’s work implies an evaluative judgment about how significant the experience of work is for someone, with an emotional as well as a cognitive component (Martela and Pessi, 2018). Meaningful work is commonly defined as work experienced as personally significant, purposeful, and contributing to the greater good (Rosso et al., 2010; Steger et al., 2012). Unlike job satisfaction or engagement, which emphasize affective or behavioral outcomes, meaningful work captures the existential alignment between one’s self-concept and one’s occupational role (Pratt and Ashforth, 2003). A multidimensional definition of work meaningfulness contends a “process” perspective to define meaningful experiences as episodic and subjective positive experiences at work while a unidimensional perspective highlights individuals’ judgment that they have a “meaningful work” that helps them accomplish significant and valuable goals aligned with their personal existential values (Allan et al., 2015; Allan et al., 2019). Most researchers agree on the former conceptualization of meaningful work as a multidimensional, subjective, evaluative, and psychological construct rather than an objective part of any work (Martela and Pessi, 2018). A multidimensional conceptualization implies that there are multiple sources of meaningfulness experiences instead of a single or few causes. Theorists have identified several dimensions of meaningful work, including positive meaning, meaning-making through work, and the motivation to contribute beyond the self (Steger et al., 2012), as well as broader thematic pathways such as authenticity, unity, and service (Lips-Wiersma and Morris, 2009) and self-connection, contribution, unification, and individuation (Rosso et al., 2010). A comprehensive measure of meaningful work identified four subdimensions, including “unity with others,” “developing the inner self,” “serving others,” and “expressing full potential” (Lips-Wiersma and Wright, 2012). In their comprehensive review of the literature looking for a definitive meaning of “work meaningfulness,” Martela and Pessi (2018) identified three common elements that have been emphasized in 36 separate definitions of work meaningfulness, namely significance, broader purpose, and self-realization. Significance refers to the intrinsic value embedded in one’s work-related activities, and the degree of significance assigned to one’s work contributes to answering the existential question of “why” one continues engage in such work, beyond instrumental necessity. Broader purpose is connected to the idea of doing something larger than those serving one’s individual benefits and contributing to the society. Finally, self-realization is the feeling of connectedness between oneself and work, which is the opposite of feelings of alienation from one’ work that arises when individuals cannot express themselves or exert control on aspects of their work (Tummers and Knies, 2013). A meaningful work provides opportunities for growth and facilitate individuals to realize their full potential (Martela and Pessi, 2018). A qualitative review has identified 28 different measurement scales for operationalizing meaningful work, pointing to either an absence of scholarly consensus on the construct or diversity of perspectives on it (Lozovetska et al., 2024).

Empirical evidence provides support for the distinctiveness and predictive validity of meaningful work construct, with studies demonstrating that meaningful work is positively associated with life satisfaction, work engagement, organizational commitment, and ethical behavior, while negatively associated with burnout and turnover intention (Allan et al., 2016; Fairlie, 2011; Lysova et al., 2019). Empirical evidence has demonstrated several outcomes for meaningful work, including work-related, performance-related, and individual outcomes that include but are not limited to higher levels of engagement, job satisfaction, life satisfaction, commitment, creativity, knowledge sharing, and lower intentions to quit (Allan et al., 2015; Allan et al., 2019; Duffy et al., 2014; Geldenhuys et al., 2014). Additionally, the experience of meaningful work has been theoretically grounded in several foundational frameworks that explain why individuals perceive their work as purposeful and significant. In a comprehensive meta-analysis of work meaningfulness outcomes (Allan et al., 2019), researchers have used job characteristics theory as a framework to conceptualize the effects of meaningful work on work-related outcomes such as job satisfaction, engagement, commitment, work performance and turnover intentions, claiming that high core job dimensions of task significance, task identity and autonomy are associated with greater meaningfulness. Researchers have also utilized self-determination theory in explaining meaningful work whereby people perceive their work to be significant and valuable when it provides them with enhanced autonomy, competence, and relatedness (Bailey et al., 2018). Work has also been associated with greater meaningfulness from an identity theory perspective, particularly when it aligned with one’s self concept (Pratt and Ashforth, 2003). Work meaningfulness perceptions are influenced by a plethora of factors from job design to management style, and it then has a positive influence on a range of individual and organizational outcomes (Lysova et al., 2019). Moreover, job design features such as autonomy and task significance (Hackman and Oldham, 1976), leadership styles (e.g., servant or ethical leadership), and organizational justice climate have been identified as key antecedents (Rosso et al., 2010). The factors that lead to higher perceptions of work meaningfulness include job design whereby aspects of job characteristics such as work-role fit and job enrichment have been found to contribute to enhanced meaningfulness; leadership styles such as transformational leadership and leader-member exchange that correlate with higher perceived meaningfulness; organizational and workplace related factors such as a supportive culture and positive workplace climate that increase employees’ sense of meaningfulness (Lozovetska et al., 2024).

Collectively, these theoretical underpinnings suggests that meaningful work is not only a desirable individual experience but also a strategic organizational asset that enhances both human sustainability and long-term performance. Previous research has also evidenced the significant role of supportive unions in fostering positive employee attitudes toward work and the significance of “union premium” in fulfillment of a sense of meaningfulness at work. To our knowledge, this connection has not been previously tested in a non-western emerging country context with a large sample representative of public employees, along with the potential roles of other wellbeing variables.

## 3 A brief overview of unionization among civil servants in Turkiye

Although the organization of civil servants in Turkey dates back to earlier years, genuine unionization only became possible in the aftermath of the 1961 Constitution. Adopting the principle of the social state, the 1961 Constitution granted all employees the right to unionize and provided constitutional protection for union rights through Article 46. Four years later, the State Personnel Unions Act (Law No. 624, 1965) regulated the unionization rights of civil servants. While recognizing the right to union organization, the law also imposed significant restrictions, explicitly banning meetings, demonstrations, and strikes (Çelik, 2014).

Currently, civil servants’ unions in Turkey are organized into eleven service branches defined by Law No. 4688 (Official Gazette, 2001). This framework permits multiple unions with different ideological or political orientations to operate within the same branch. As of July 2025, there were 277 registered civil servants’ unions distributed across various branches, from office, banking, and insurance services to education, training, and science, as well as health and social services and press and communication. This fragmented structure is also reflected at the confederation level, where 18 civil servants’ confederations are active nationwide as of July 2025.

According to the 2025 data (see Appendix Table 1 for a detailed account of membership data), Memur-Sen, with approximately 1.079 million members, ranks first in 10 out of 11 service branches and is the confederation with the largest membership. Türkiye Kamu-Sen follows in second place with 560,000 members, while Birleşik Kamu-İş ranks third with 189,000 members, and KESK fourth with 166,000 members. According to the 2025 figures of the Ministry of Labor, out of a total of 3.016 million public employees, 2.019 million are unionized, corresponding to a unionization rate of 76.88% (compared to 47.94% in 2002).

Following the adoption of Law No. 4688, the trajectory of civil servants’ union membership in Turkey between 2002 and 2025 reveals a strikingly asymmetric pattern. During this 23-year period of uninterrupted single-party government, the membership of the three major confederations evolved in very different directions. The Confederation of Public Employees’ Trade Unions (KESK) experienced a decline of 36.6 percent, while the Confederation of Turkish Public Workers’ Unions (Türkiye Kamu-Sen) grew by about 70 percent. The most extraordinary development, however, was the unprecedented rise of the Confederation of Civil Servants’ Trade Unions (Memur-Sen). Starting with just 41,871 members in 2002, Memur-Sen expanded to nearly 1.8 million members by 2025, marking a staggering increase of more than 2,400 percent. By 2025, out of the 2.019 million unionized civil servants in Turkey, approximately 1.805 million, nearly 78 percent were affiliated with Memur-Sen.

Although this might initially suggest remarkable organizational success, such an extraordinary concentration of membership warrants critical examination. The sharp rise in public sector unionization overall, combined with the overwhelming dominance of a single confederation known to be aligned with the ruling party, raises important questions about the underlying drivers of union affiliation in the Turkish public sector.

In theory, a more consolidated union structure could be seen as strengthening collective bargaining power. In practice, however, the strength of Turkish civil servants’ unions appears to derive less from their members’ solidarity than from their proximity to political authority. As noted by earlier studies (Çelik, 2014; Uçkan Hekimler, 2023), this has been perceived as an organizational achievement, yet it comes at the cost of undermining individual union freedom. Many civil servants report that they view their union as closely tied to a political party (Özaydın and Han, 2014), and a significant number strategically affiliate with “politically acceptable” unions to secure promotions or favorable transfers.

In light of the previous evidence, we examine union membership and perceived union support as antecedents to work meaningfulness and subsequent wellbeing outcomes. We also examine the roles of status anxiety and job stress as two negative correlates to test potential interaction effects with union membership and support, investigating whether union involvement can mitigate the detrimental effects of workplace stressors on meaningfulness and wellbeing.

## 4 Union membership and union support on perceived work meaningfulness and wellbeing outcomes

Perceived union support (PUS) is usually conceptualized as an extension of perceived organizational support (POS) (Eisenberger et al., 2001; Tetrick et al., 2007). However, unions are qualitatively different from employing organizations as they are member-driven associations with instrumental, expressive, and political dimensions (Heery and Kelly, 1994). From this perspective, PUS cannot be reduced to a unidimensional perception of care. Instead, it reflects both instrumental support (bargaining for wages, job security, protections) and expressive support (providing solidarity, identity, and a collective voice). This dual function makes unions distinct institutional actors in shaping work meaningfulness, particularly in politicized public-sector contexts. From a theoretical standpoint, PUS can be positioned as a multi-level resource that enriches individual work experiences through three interlocking mechanisms. First, self-determination theory (SDT) suggests that unions contribute to the satisfaction of autonomy (voice and procedural protections), competence (advocacy for fair treatment and resources), and relatedness (membership in a collective) (Ryan and Deci, 2017). Second, drawing on job characteristics theory (Hackman and Oldham, 1976), unions enhance perceptions of task significance and fairness, thereby increasing the sense that one’s work matters. Finally, social exchange theory (Blau, 1964; Cropanzano and Mitchell, 2005) highlights that supportive unions generate reciprocity norms, encouraging employees to internalize more positive work attitudes.

Based on these frameworks, we propose that PUS acts as a proximal driver of work meaningfulness because it simultaneously addresses individual needs, enriches job design, and fosters reciprocal attachments. By contrast, formal membership without perceived support merely confers an institutional label but lacks the psychological resources that create meaning. This distinction clarifies why empirical results often show mixed associations between union membership and well-being (Borjas, 1979; Blanchflower and Bryson, 2020a), but more consistent effects for perceptions of support.

Existing research on the relationship between union membership and job satisfaction has produced conflicting findings, with some studies reporting a negative association (Borjas, 1979; García-Serrano, 2009) and others finding a positive link (Blanchflower and Bryson, 2020a; Blanchflower and Bryson, 2020b; Blanchflower et al., 2021; Flavin and Shufeldt, 2016). However, the literature has primarily focused on the general measure of job satisfaction, overlooking the potential impact of unionization on the specific dimension of perceived work meaningfulness. Interestingly, the degree to which employees believe their union is supportive of their interests and advocates for their well-being may be a critical factor in determining their perceptions of work meaningfulness in addition to satisfaction with one’s job (Davis, 2012).

**Figure 1:**
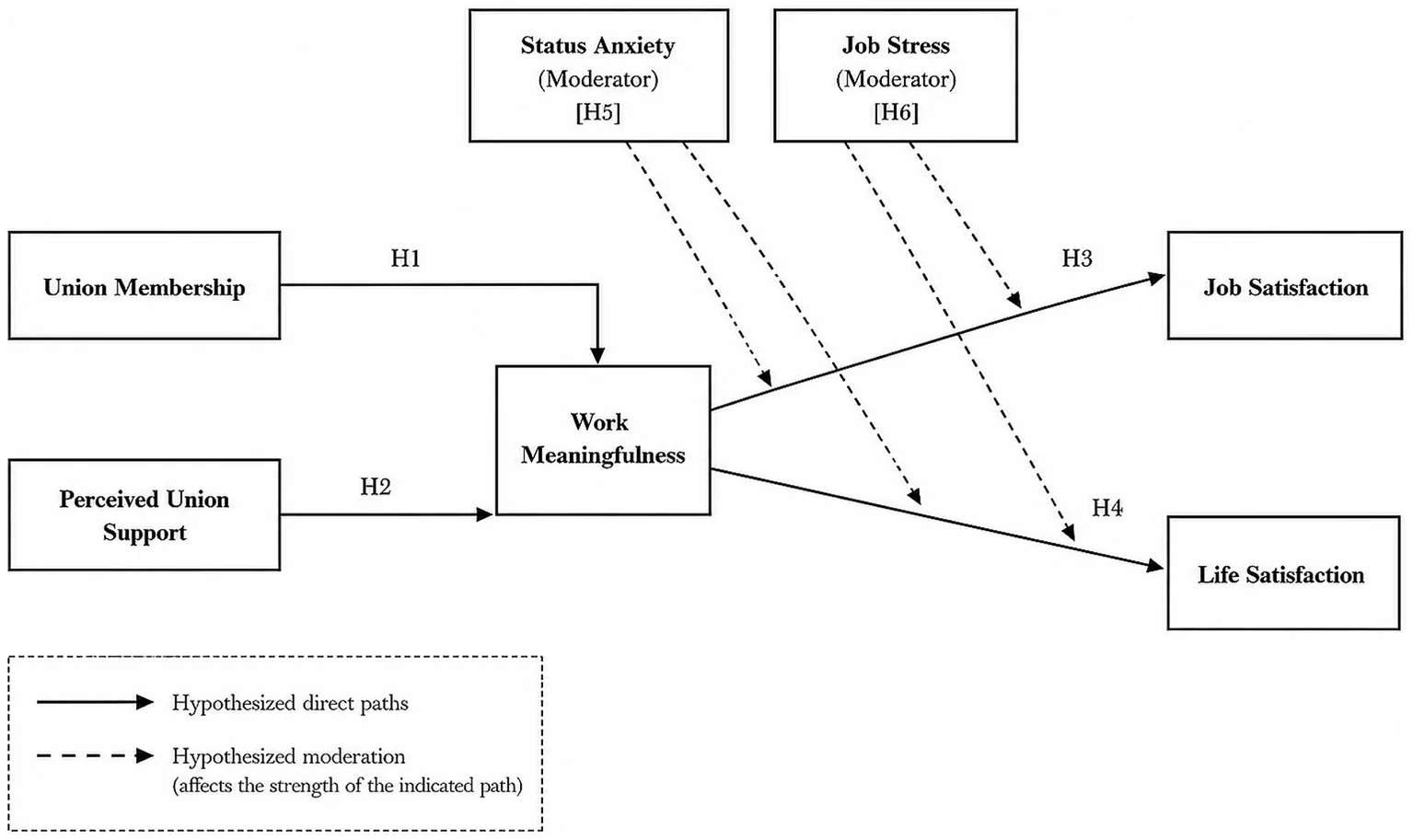
Conceptual model of the hypothesized relationships.

Building on prior research that has explored the multifaceted effects of unionization on various aspects of work attitudes and experiences (Davis, 2012; Flavin and Shufeldt, 2016; Tummers and Knies, 2013) we hypothesize that union membership, and union support beyond that will have a positive association with perceived work meaningfulness. Employees often rely on unions to address unsatisfactory work conditions, enhance their work life through increased compensation, benefits, and improved working environments, and safeguard against unfair treatment. From a self-determination theory (Ryan and Deci, 2017) perspective, meaningful work emerges when employees experience autonomy, competence, and relatedness in their work roles through such supportive union actions. Through these mechanisms, unions can shape not only employees’ evaluations of their jobs but also their perceptions that their work is significant, purposeful, and connected to broader social goals. Consequently, unions can exert a significant influence on their members’ work experiences (Blanchflower and Bryson, 2020a) and perceptions, including their sense of work meaningfulness. We anticipate differences in perceived work meaningfulness between unionized and non-unionized public sector employees. Unionized employees may report higher levels of meaningfulness given the pivotal role unions play in advocating for improved working conditions, job security, and compensation. These tangible benefits can foster a greater sense of purpose, contribution, and value at work. However, simple membership alone may not be sufficient to generate meaningfulness; rather, it is the strength of perceived union support that is more likely to explain outcomes such as job satisfaction. Work meaningfulness is expected to serve as a facilitating mechanism in this process, linking perceived union support to broader indicators of employee well-being (see Figure 1).

The Turkish public sector context where union membership may sometimes be driven by instrumental considerations such as career advancement, organizational influence, or political alignment rather than identification with collective representation, introduces a critical complication. These dynamics could weaken the relationship between formal membership and employees’ subjective experiences of work. Nevertheless, union membership remains the primary institutional channel through which employees gain access to representation, collective voice, legal protections, and various resources. Even when membership decisions are partly instrumental, membership may still expose employees to workplace support structures and collective identities that might contribute to a stronger sense of purpose and significance. As such, we retain the expectation that union membership will be positively associated with perceived work meaningfulness, while recognizing that the strength of this relationship may depend on whether membership is accompanied by perceptions of genuine union support. That is, formal membership may provide an initial basis for meaningfulness through access to collective representation, while perceived union support has the potential to explain additional variance beyond membership status alone. Thus;

*H1*: Union membership is positively associated with perceived work meaningfulness among public sector employees.

*H2*: Perceived union support is positively associated with work meaningfulness and job satisfaction above and beyond union membership.

However, some workers, particularly those who prioritize intrinsic job characteristics and professional development opportunities over extrinsic factors, may not experience the same boost in their perceived work meaningfulness. These individuals may be more focused on aspects such as autonomy, significance, and competence, which may be less directly impacted by union representation and activities. Ultimately, the relationship between union membership and perceived work meaningfulness is likely to be nuanced and influenced by individual differences in work values and priorities.

*H3*: Work meaningfulness mediates the positive relationship between perceived union support and job satisfaction.

*H4*: Work meaningfulness mediates the positive relationship between perceived union support and life satisfaction.

## 5 The roles of status anxiety and job stress

Trade unions represent a unique form of institutionalized social support that can provide both instrumental and emotional resources to workers. Trade unions create protective mechanisms through higher wage and benefit standards, working hours limits, workplace hazards protections, while also promoting well-being by encouraging democratic participation and a sense of community among workers. Research demonstrates that unions raise wages, decrease inequality, reduce discrimination, and affect multiple determinants of health while improving workplace conditions. Unions provide both collective bargaining power to address systemic stressors and peer support networks that can help individuals cope with personal anxieties about career advancement and workplace pressures, potentially preserving workers’ sense that their work has meaning even under stressful conditions (Feiveson, 2023; Hagedorn et al., 2016; Leigh and Chakalov, 2021).

In contexts where unions are closely aligned with political power, employees may experience status anxiety, reflecting concerns about social standing, favoritism, or fear of exclusion if not affiliated with the “right” union. Such anxieties can erode the positive effects of PUS by undermining employees’ sense of authenticity and security at work.

Status anxiety represents individuals’ fears about their relative position in social hierarchies and concerns about maintaining or improving their standing (De Botton, 2004; Wilkinson and Pickett, 2017). This form of anxiety can undermine work meaningfulness through several mechanisms. First, status-anxious individuals tend to focus on extrinsic motivators (salary, prestige, advancement) rather than intrinsic aspects of work that typically contribute to meaningfulness, such as personal growth, contribution to others, and alignment with values (Kasser and Ryan, 1996). Second, chronic concerns about one’s position relative to others can create a comparative mindset that diminishes appreciation for the inherent value of one’s work contributions. Third, status anxiety is associated with increased stress and negative affect, which can impair the cognitive-emotional processes through which individuals derive meaning from their work experiences. Research on materialism and wellbeing supports this proposition, showing that individuals who prioritize status-related goals report lower levels of meaning and purpose in life (Dittmar et al., 2014). In organizational contexts, employees who are preoccupied with hierarchical positioning may struggle to connect with the deeper purpose of their work role. Status anxiety might attenuate the positive association of meaningful work with positive outcomes and exacerbate the dark side of meaningfulness. Thus,

*H5*: Status anxiety is negatively associated with perceived work meaningfulness, job satisfaction and life satisfaction. Status anxiety moderates the relationship between work meaningfulness and job and life satisfaction such that the indirect effect of perceived union support through meaningfulness varies across levels of status anxiety.

From a job demands- resources (JD-R) perspective, high levels of job stress pose a job demand which might lead to negative work-related and personal outcomes, especially when employees do not have adequate resources to buffer against their deleterious effects (Bakker and Demerouti, 2017). Job stress could be a result of multiple work-related factors or work stressors, including but not limited to role conflicts, too much work, time pressures, lack of job security, organizational politics, and lack of effective consultation (Frone et al., 1995). When there is a lack of balance between job demands and resources in driving positive employee outcomes, negative experiences such as decreased meaningfulness perceptions are likely to arise. Elevated levels of job stress have previously been investigated as a significant barrier against positive work outcomes such as commitment, engagement, satisfaction, and innovative work behavior (Lazarus and Folkman, 1984; Phanniphong et al., 2024; Tims et al., 2013). In line with this stream of research, we argue that high levels of job stress can attenuate the positive effects of union support on work meaningfulness by reducing individuals’ capacity to nurture feelings of significance or purpose and by keeping them occupied with daily hustles instead of reaching for higher order feelings like transcendentalism. The positive effects of perceived union support might be eliminated or weakened under the pressure created by job stress. Thus;

*H6*: Job stress is negatively associated with perceived work meaningfulness, job satisfaction and life satisfaction. Job stress moderates the relationship between work meaningfulness and job and life satisfaction such that the indirect effect of perceived union support through meaningfulness varies across levels of job stress.

## 6 Materials and methods

### 6.1 Participants and procedure

We collected data during a 5-month period, from February to June 2025, among a large sample of public employees working across Turkey. Our data collection request was approved by the Ethics and Research Committee of the corresponding author’s university. We sent out our survey link through Electronic Information Management System, a special intranet for public organizations countrywide, to all the public agencies to be filled by any working adult of any status in the organization. We provided a brief explanation of our research purpose at the beginning of the survey and added an informed consent box. The participation in our study was completely on a voluntary basis with full anonymity. A total of 1,673 participants provided full response to our questionnaire.

## 7 Measures

### 7.1 Satisfaction with life scale

The level of life satisfaction was assessed using the Satisfaction with Life Scale, developed by Diener et al. (1985) and adapted into Turkish by Durak et al. (2010). The scale consists of five items, including statements such as “My life conditions are excellent.” Responses were rated on a 5-point Likert scale (1 = strongly disagree, 5 = strongly agree). The Cronbach’s alpha reliability coefficient of the scale in this study was calculated as 0.868.

### 7.2 Job satisfaction

The Job Satisfaction Scale developed by Brayfield and Rothe (1951) and later adapted by Judge et al. (1998) in a 5-item short form was used. Items 3 and 5 of the scale, which was adapted into Turkish by Keser and Öngen Bilir (2019), were reverse coded. The scale includes statements such as “I am quite satisfied with my current job” and “I think my job is unsatisfying” (reverse coded). The Cronbach’s alpha reliability coefficient for the scale was calculated as 0.778. Agreement with the statements was scored using a 5-point Likert-type scale.

### 7.3 Work meaningfulness

Meaningful work experiences were measured using the 21-item “Meaningful Work Scale for Educational Organizations” developed by Göçen and Terzi (2019) using items from four different scales for validity. The scale consists of 6 dimensions: items 1–5 assess meaningful job, items 6–8 measure pursuit of meaning at work, items 9–12 evaluate work relationships, items 13–15 examine transcendentalism, items 16–18 assess humility, and items 19–21 measure meaningfulness leadership. The scale demonstrated good internal consistency (*α* = 0.86).

### 7.4 Status anxiety scale

The Status Anxiety Scale developed by Day and Fiske (2016) was adapted into Turkish by Sürücü et al. (2022). Agreement with the statements in the five-item scale was scored using a 5-point Likert scale. Higher scores on participants’ responses indicate higher levels of status anxiety. The scale includes statements such as “I am very worried that I will not achieve my career goals” and “I worry that my social status will not change.” The Cronbach’s alpha reliability coefficient for the scale was calculated as 0.89.

Perceived labor union support was operationalized using a single statement asking participants to rate their perceptions of union support on a 5-point scale ranging from 1 (Lowest) to 5 (Highest).

Job stress was measured using a single item asking participants to rate their individual experiences on a 3-point scale ranging from 1 (Lowest) to 3 (Highest). Although multi-item scales are generally preferred, single-item measures of perceived stress have been widely employed in large organizational and occupational health surveys where questionnaire length and respondent burden are important considerations (Elo et al., 2003; Dolbier et al., 2005). Similarly, recent research on a large sample representative of France, the USA and Canada employed a single-item measure of union experience based on the question, *“On the whole, how would you describe your experience with union membership?”* on a five-point Likert scale (De La Haye et al., 2026).

Trade union membership was assessed using a yes/no question, and participants who responded positively were asked to select from a list of existing confederations to verify their membership status.

We also examined the frequency of interaction with the union, using the item “How often do you engage with your trade union?” rated on a 5-point scale ranging from 1 (Never) to 5 (Always).

## 8 Data analysis

All hypothesis tests were conducted using Jamovi and JASP (for Hayes’s process macro), both R-based open-source statistical analysis tools. We first examined construct validity with confirmatory factor analysis. Next, bivariate associations were inspected. Primary tests used multiple regression to explain perceived meaningfulness (controls included demographics and pay/fit variables), and two mediation models tested whether meaningfulness mediated the effect of perceived union support on (a) job satisfaction and (b) life satisfaction. Moderation models examined whether status anxiety and job stress moderated the perceived union support (PUS) → meaningfulness → job satisfaction/life satisfaction paths. Indirect effects were estimated with bootstrapped confidence intervals (1,000 samples).

There was no missing data as the survey link required all items to be filled before hitting the submit button. We examined the normal distribution with skewness and kurtosis with a cutoff value of +/− 3 and found all constructs to be appropriate. We used maximum likelihood estimation for model testing. We tested for chi-squared statistics, RMSEA (<0.06), CFI (>0.95), and TLI (0.95) for confirming model fit (Hu and Bentler, 1999). The confirmatory factor analysis findings for the four scales are presented in Table 1.

**Table 1:** Confirmatory factor analysis.

Variable	*X*^2^/df	CFI	TLI	RMSEA
Status anxiety	8.73/3	0.99	0.99	0.033
Meaningfulness	1215/174	0.95	0.93	0.06
Job satisfaction	11.1/4	0.99	0.99	0.032
Life satisfaction	13.2/3	0.99	0.98	0.045

The descriptive analyses reported in Table 2 were conducted using the full sample of 1,673 respondents. However, because perceived union support can only be meaningfully assessed among individuals with union affiliation, the hypothesis testing analyses involving perceived union support were restricted to respondents who reported union membership and provided valid responses to the union support measure (*n* = 1,291). The mediation and moderated mediation analyses presented in Tables 3, 4 are based on this subsample. This should be considered a boundary condition when interpreting the findings, as the reported relationships pertain specifically to unionized employees.

**Table 2:** Descriptives and inter-correlations for all variables.

Variables	M	(SD)	1	1a	1b	1c	1d	1e	1f	2	3	4	5	6	7	8	9
1. Meaningfulness	3.54	0.51	(0.86)														
1a. Meaningful job	3.52	0.91	0.778^***^	(0.89)													
1b. Pursuit of meaning	2.98	0.93	0.249^***^	−0.097^***^	(0.84)												
1c. Work relationships	3.55	0.83	0.678^***^	0.374^***^	0.008	(0.88)											
1d. Transcendentalism	3.53	0.86	0.751^***^	0.662^***^	−0.026	0.377^***^	(0.82)										
1e. Humility	4.01	0.71	0.483^***^	0.254^***^	0.013	0.198^***^	0.251^***^	(0.73)									
1 f. Meaningful leadership	3.68	0.71	0.654^***^	0.319^***^	0.159^***^	0.430^***^	0.394^***^	0.283^***^	(0.80)								
2. Status anxiety	2.85	1.11	−0.255^***^	−0.346^***^	0.190^***^	−0.213^***^	−0.211^***^	−0.140^***^	−0.086^***^	(0.92)							
3. Job satisfaction	3.62	0.68	0.417^***^	0.502^***^	−0.209^***^	0.299^***^	0.391^***^	0.209^***^	0.180^***^	−0.347^***^	(0.71)						
4. Life satisfaction	3.03	0.49	0.237^***^	0.262^***^	−0.060^*^	0.186^***^	0.201^***^	0.067^**^	0.126^***^	−0.312^***^	0.292^***^	(0.75)					
5. How much do you love your job? (1: none, 5: very much)	3.99	1.03	0.515^***^	0.617^***^	−0.182^***^	0.363^***^	0.520^***^	0.159^***^	0.193^***^	−0.342^***^	0.530^***^	0.243^***^					
6. Are you satisfied with your current socioeconomic status? (1: none, 5: very much)	3.08	1.11	0.311^***^	0.353^***^	*0.069^**^	0.232^***^	0.259^***^	0.108^***^	0.138^***^	−0.483^***^	0.287^***^	0.429^***^	0.397^***^				
7. Job stress level (3:high, 1:low)	2.14	0.64	−0.149^***^	−0.171^***^	0.157^***^	−0.183^***^	−0.142^***^	−0.077^***^	−0.065^***^	0.313^***^	−0.233^***^	−0.195^***^	−0.210^***^	−0.256^***^			
8. Perceived union support	2.61	1.30	0.296^***^	0.281^***^	−0.027	0.240^***^	0.263^***^	0.088^**^	0.192^***^	−0.191^***^	0.173^***^	0.129^***^	0.232^***^	0.236^***^	−0.099^***^		
9. Experience	11.0	9.32	0.097^***^	0.102^***^	−0.109^***^	0.074^**^	0.125^***^	0.090^***^	0.065^**^	−0.060^*^	0.085^***^	−0.001	0.121^***^	0.092^***^	−0.052^*^	0.159^***^	
10. Age	41.2	9.11	0.086^***^	0.084^***^	−0.137^***^	0.055^*^	0.145^***^	0.102^***^	0.078^**^	−0.086^***^	0.081^***^	−0.046	0.119^***^	0.096^***^	−0.094^***^	0.196^***^	0.666^***^

^***^ *p* < 0.001, **p < 0.01, ^*^p < 0.05, Numbers in parentheses shows Cronbach’s alpha values.

**Table 3:** Work meaningfulness mediates the positive relationship between perceived union support and job satisfaction.

				95% CI			
Effect		B	*SE*	*LL*	*UL*	*β*	*p*
Indirect	Labor union membership ⇒ meaningfulness ⇒ JobSat	−0.031	0.259	−0.539	0.476	−0.001	0.903
	Perceived union support ⇒ meaningfulness ⇒ JobSat	0.062	0.006	0.048	0.075	0.118	**< 0.001**
Component	Labor union membership ⇒ meaningfulness	−0.061	0.503	−1.048	0.924	−0.003	0.903
	Meaningfulness ⇒ JobSat	0.512	0.034	0.448	0.582	0.398	**< 0.001**
	Perceived union support ⇒ meaningfulness	0.120	0.011	0.099	0.141	0.296	**< 0.001**
Direct	Labor union membership ⇒ JobSat	0.281	0.619	−0.932	1.494	0.454	0.650
	Perceived union support ⇒ JobSat	0.028	0.014	0.001	0.056	0.054	**0.038**
Total	Labor union membership ⇒ JobSat	0.249	0.671	−1.067	1.565	0.010	0.711
	Perceived union support ⇒ JobSat	0.090	0.014	0.062	0.119	0.172	**< 0.001**

*N* = 1,291. Betas are completely standardized effect sizes. Statistically significant values are indicated in bold.

**Table 4:** Work meaningfulness mediates the positive relationship between perceived union support and life satisfaction.

				95% CI			
Effect		B	*SE*	*LL*	*UL*	*β*	*p*
Indirect	Labor union membership ⇒ meaningfulness ⇒ LifeSat	−0.012	0.099	−0.206	0.182	−0.012	0.903
	Perceived union support ⇒ meaningfulness ⇒ LifeSat	0.023	0.003	0.016	0.031	0.062	**< 0.001**
Component	Labor union membership ⇒ meaningfulness	−0.061	0.503	−1.048	0.924	−0.003	0.903
	Meaningfulness ⇒ LifeSat	0.196	0.026	0.144	0.248	0.209	**< 0.001**
	Perceived union support ⇒ meaningfulness	0.120	0.011	0.099	0.141	0.296	**< 0.001**
Direct	Labor union membership ⇒ LifeSat	0.722	0.480	−0.219	1.664	0.040	0.133
	Perceived union support ⇒ LifeSat	0.025	0.010	0.003	0.046	0.065	**0.020**
Total	Labor union membership ⇒ LifeSat	0.710	0.491	−0.251	1.673	0.039	0.148
	Perceived union support ⇒ LifeSat	0.048	0.010	0.028	0.069	0.127	**< 0.001**

*N* = 1291. Statistically significant values are indicated in bold.

## 9 Reliability and validity

Following the CFA analysis, we assessed the composite reliability of the meaningfulness factors, job satisfaction, life satisfaction and status anxiety. Internal consistency reliability for all subscales was calculated using Cronbach’s alpha and with all coefficients exceeding 0.70. The overall models demonstrated good internal consistency: Total meaningfulness (*α* = 0.86), Meaningful job (*α* = 0.89), Pursuit of meaning (*α* = 0.84), Work relationships (*α* = 0.88), Transcendentalism (*α* = 0.82), Humility (*α* = 0.73), Meaningful leadership (*α* = 0.80), Status anxiety (*α* = 0.92), Job satisfaction (*α* = 0.71), and Life satisfaction (*α* = 0.75). These values meet conventional thresholds for research use.

## 10 Findings

A2 in Appendix summarizes the demographic profile of the sample (*N* = 1,673). Participants’ mean age was 41.2 years (SD = 9.11), with an average of 11 years of work experience (SD = 9.32). The gender distribution indicated a slightly higher proportion of men (58.2%) than women (41.8%). Most participants held at least a bachelor’s degree (53.7%), with 25.9% completing a master’s or doctoral degree. In terms of marital status, the majority were married (75.3%), while 24.7% were single. Approximately one-third of respondents had no children (31.1%), whereas 30.8% had two children, yielding an average of 1.36 children (SD = 1.17). Regarding income, 31.1% reported monthly earnings between 50,000–70,000 TL (around 1700 US dollars per month), and 24.5% between 30,000–50,000 TL (around 1200 US dollars per month). Administrative roles were relatively limited, with 26.7% reporting no managerial or leadership positions, while more than half (55.5%) indicated “other” roles. Importantly, 77.2% of respondents reported union membership, underscoring the highly unionized character of the sample which reflects the overall public sector unionization rates across the country (76.8%, as discussed in the above section).

Union affiliation patterns revealed that Memur-Sen was the dominant confederation, with 44.7% of unionized respondents reporting membership. Türkiye Kamu-Sen (19.1%) and “Other” smaller confederations (24.4%) followed. However, it should be noted that the questionnaire included all available confederation options, and responses categorized as “other” may reflect participants’ lack of knowledge regarding the confederation affiliation of their specific unions. Birleşik Kamu-İş (1.8%), KESK (2.6%), and independent unions (2.2%) represented smaller proportions. Other confederations (e.g., Hür-Sen, Hak-Sen, Tüm Memur-Sen) accounted for less than 1% each. These figures closely align with official statistics, confirming the organizational dominance of Memur-Sen among Turkish civil servants.

Perceived support from trade unions was relatively low overall (M = 2.61). As presented in Table 5, 27.5% rated their perceived support at the lowest level, and 19.2% selected level 2, while only 24.8% rated their commitment at levels 4 or 5. This indicates that although union membership is widespread, psychological attachment and loyalty to unions appear weak across the sample.

**Table 5:** Perceived union support.

**Rate on a scale of 1 to 5 your perceived level of support from the labor union (1: lowest 5: highest)**	**Counts**	**% of Total**	**Mean**
1	355	27.5%	2.61
2	248	19.2%	
3	369	28.6%	
4	191	14.8%	
5	129	10.0%	

Descriptive statistics and intercorrelations for all study variables are presented in Table 2. Perceived work meaningfulness was moderately high (M = 3.54, SD = 0.51) and showed strong positive associations with job satisfaction (*r* = 0.42, *p* < 0.001) and a smaller positive association with life satisfaction (*r* = 0.24, *p* < 0.001). Among its subdimensions, meaningful job (*r* = 0.78, *p* < 0.001), work relationships (*r* = 0.68, *p* < 0.001), transcendentalism (*r* = 0.75, *p* < 0.001), and meaningful leadership (*r* = 0.65, *p* < 0.001) exhibited the strongest correlations with overall meaningfulness. Status anxiety was negatively correlated with work meaningfulness (*r* = −0.26, *p* < 0.001), job satisfaction (*r* = −0.35, *p* < 0.001), and life satisfaction (*r* = −0.31, *p* < 0.001), indicating its detrimental role. Socioeconomic satisfaction (M = 3.08, SD = 1.11) was positively related to work meaningfulness (*r* = 0.31, *p* < 0.001) and job satisfaction (*r* = 0.29, *p* < 0.001), while negatively related to status anxiety (*r* = −0.48, *p* < 0.001). Perceived union support was positively correlated with work meaningfulness (*r* = 0.29, *p* < 0.001), job satisfaction (*r* = 0.17, *p* < 0.001) and life satisfaction (*r* = 0.12, *p* < 0.001) while it negatively associated with status anxiety (*r* = −0.19, *p* < 0.001). Job stress levels negatively associated with work meaningfulness (*r* = −0.14, *p* < 0.001), job satisfaction (*r* = −0.23, *p* < 0.001) and life satisfaction (*r* = −0.19, *p* < 0.001) while it positively associated with status anxiety (*r* = 0.31, *p* < 0.001).

Regression analysis on Table 6 demonstrated that perceived union support (B = 0.050, *p* < 0.001), socioeconomic satisfaction (B = 0.027, *p* = 0.030), loving one’s job (B = 0.114, *p* < 0.001), and income–expectation fit (B = 0.044, *p* = 0.012) were significant correlates of meaningfulness. Higher monthly income levels were negatively associated with meaningfulness compared to the lowest income group (*p*s < 0.05). One possible explanation for this counterintuitive finding between higher income categories and meaningfulness is that higher-income positions in public organizations often involve increased administrative responsibilities and reduced direct service engagement. This might potentially weaken employees’ perceptions of social contribution and purpose. Alternatively, the finding may reflect the distinction between extrinsic rewards and intrinsic sources of meaning emphasized in meaningful-work research (Kasser and Ryan, 1996). Because income was included only as a control variable, these interpretations remain speculative and warrant future investigation. The overall model explained 34% of the variance in meaningfulness, highlighting the relative importance of subjective perceptions (union support, socioeconomic satisfaction, job love) over demographic variables.

**Table 6:** Effect of antecedent variables on work meaningfulness.

Variable	Unstandardized Coefficients	*t*	*p*
	B		
(Constant)		3.836	**<0.001**
Labor union membership (member-non*member)	0.296	0.689	0.491
Perceived support from the union (1: lowest, 5: highest)	0.050	4.003	**<0.001**
Satisfaction with current socioeconomic status (1: lowest, 5: highest)	0.027	2.169	**0.030**
Fit between education and job (yes-no)	0.049	1.771	0.077
How much do you love your job? (1: none at all, 5: very much)	0.114	6.647	**<0.001**
Fit between income and expectation from work	0.044	2.551	**0.012**
Fair pay perception (no-yes)	0.006	0.238	0.812
Education			
1–0	0.184	1.154	0.249
2–0	0.353	2.290	**0.022**
3–0	0.267	1.747	0.081
4–0	0.295	1.911	0.056
Marital status (0–1)	−0.054	−1.729	0.086
Gender (0–1)	0.021	0.819	0.413
Monthly income			
1–0	−0.064	−1.946	0.052
2–0	−0.105	−2.531	**0.012**
3–0	−0.145	−3.590	**<0.001**
4–0	−0.141	−2.463	**0.014**
5–0	−0.197	−2.222	**0.026**
Age	−0.000	−1.406	0.160
Frequency of interaction with the union (1: none at all, 5: very much)	−0.004	−0.237	0.813
Job stress level (3: highest, 1: lowest)			
2–3	0.017	0.604	0.546
1–3	0.047	1.162	0.245
Adj. R square	0.342		
*F*	32.7		
*p*	**<0.001**		

Statistically significant values are indicated in bold.

Mediation analysis results on Table 3 showed that perceived union support is associated with higher work meaningfulness (B = 0.120, *p* < 0.001), which in turn associated with greater job satisfaction (B = 0.512, *p* < 0.001). The indirect effect of union support on job satisfaction via meaningfulness was significant (B = 0.062, 95% CI [0.048, 0.075], p < 0.001). The direct effect of union support on job satisfaction remained small but significant (B = 0.028, *p* = 0.038), indicating partial mediation. In contrast, union membership per se had no significant effect on meaningfulness or job satisfaction.

Table 4 presents the mediation analysis for life satisfaction. Perceived union support significantly associated with work meaningfulness (B = 0.120, *p* < 0.001), which in turn was positively related to life satisfaction (B = 0.196, *p* < 0.001). The indirect effect of union support on life satisfaction through meaningfulness was significant (B = 0.023, 95% CI [0.016, 0.031], *p* < 0.001). The direct effect of union support on life satisfaction was smaller (B = 0.025, *p* = 0.020), indicating partial mediation. Again, union membership status had no significant effect on either meaningfulness or life satisfaction.

A conditional process model (PROCESS Model 14 on Table 7) tested whether status anxiety moderated the indirect effect of perceived union support (PUS) on job and life satisfaction through meaningfulness. PUS significantly associated with meaningfulness (B = 0.120, SE = 0.011, 95% CI [0.098, 0.143], *p* < 0.001). Meaningfulness is linked to job satisfaction (B = 0.442, SE = 0.034, 95% CI [0.362, 0.527], *p* < 0.001) and life satisfaction (B = 0.140, SE = 0.026, 95% CI [0.092, 0.190], *p* < 0.001). However, the interaction terms (Meaningfulness × Status Anxiety) were not significant for either outcome (*p* = 0.489, 0.307). Conditional indirect effects of PUS were significant at both low and high-status anxiety (job satisfaction: B = 0.056–0.051, *p*s < 0.001; life satisfaction: B = 0.020–0.014, *p*s ≤ 0.001). The index of moderated mediation was nonsignificant (IMM = −0.002, 95% CI [−0.010, 0.006] for job satisfaction; IMM = −0.002, 95% CI [−0.007, 0.022] for life satisfaction). These results indicate that although status anxiety was associated with lower well-being overall, it did not moderate the mediated relationship, hence contradicting H5.

**Table 7:** Summary of conditional indirect effects: the moderating role of status anxiety.

Paths and effects	B	SE	95% CI (LL – UL)	p
a path				
Perceived union support → meaningfulness	0.120	0.011	[0.098, 0.143]	<0.001
b path				
Meaningfulness → job satisfaction	0.442	0.034	[0.362, 0.527]	< 0.001
Meaningfulness × StatusA → job satisfaction	−0.017	0.024	[−0.084, 0.048]	0.489
c’ path (direct effect)				
Perceived union support → job satisfaction	0.013	0.014	[−0.015, 0.037]	0.355
Conditional indirect effects	0.056	0.007	[0.040, 0.074]	<0.001
Lower status anxiety: perceived union support × StatusA → job satisfaction	0.051	0.007	[0.036, 0.068]	<0.001
Index of moderated mediation (IMM)	−0.002	0.003	[−0.010, 0.006]	0.490
a path				
Perceived union support → meaningfulness	0.120	0.011	[0.098, 0.143]	<0.001
b path				
Meaningfulness → life satisfaction	0.140	0.026	[0.092, 0.190]	<0.001
Meaningfulness × StatusA → life satisfaction	−0.019	0.019	[−0.058, 0.017]	0.307
c’ path (direct effect)				
Perceived union support → life satisfaction	0.013	0.011	[−0.008, 0.035]	0.220
Conditional indirect effects				
Lower status anxiety: perceived union support × StatusA → life satisfaction	0.020	0.005	[0.011, 0.029]	<0.001
Higher status anxiety: Perceived union support × StatusA → life satisfaction	0.014	0.004	[0.007, 0.022]	0.001
Index of moderated mediation (IMM)	−0.002	0.002	[−0.007, 0.022]	0.309

Unstandardized coefficients (b), bias-corrected bootstrap 95% CIs (1,000 samples), SE, standard error. Conditional indirects probe mediation at low (16th percentile) and high (84th percentile) levels of StatusA; IMM shows whether the indirect effect significantly varies with the moderator (Hayes, 2015).

We next examined whether job stress moderated the indirect effect of PUS via meaningfulness (Table 8). Results again showed a strong a path (B = 0.120, SE = 0.011, 95% CI [0.097, 0.141], *p* < 0.001) and b paths to job satisfaction (B = 0.486, SE = 0.034, *p* < 0.001) and life satisfaction (B = 0.176, SE = 0.027, *p* < 0.001). Interaction terms (Meaningfulness × Job Stress) were nonsignificant (*p*s > 0.40). Conditional indirect effects remained significant at both low and high job stress (job satisfaction: B = 0.059–0.054, *p*s < 0.001; life satisfaction: B = 0.022–0.018, *p*s < 0.001). The IMM was nonsignificant (job satisfaction IMM = −0.005, 95% CI [−0.020, 0.010]; life satisfaction IMM = −0.003, 95% CI [−0.012, 0.004]). Thus, job stress did not condition the mediation, contradicting H6.

**Table 8:** Summary of conditional indirect effects: the moderating role of job stress.

Paths and effects	B	SE	95% CI (LL – UL)	*p*
a path				
Perceived union support → meaningfulness	0.120	0.011	[0.097, 0.141]	<0.001
b path				
Meaningfulness → job satisfaction	0.486	0.034	[0.402, 0.569]	< 0.001
Meaningfulness × JobStress → job satisfaction	−0.039	0.047	[−0.170, 0.088]	0.407
c’ path (direct effect)				
Perceived union support → job satisfaction	0.024	0.014	[−0.002, 0.051]	0.078
Conditional indirect effects				
Lower job stress: perceived union support × JobStress → job satisfaction	0.059	0.007	[0.045, 0.073]	<0.001
Higher job stress: perceived union support × JobStress → job satisfaction	0.054	0.008	[0.035, 0.073]	<0.001
Index of moderated mediation (IMM)	−0.005	0.006	[−0.020, 0.010]	0.409
a path				
Perceived union support → meaningfulness	0.120	0.011	[0.099, 0.142]	<0.001
b path				
Meaningfulness → life satisfaction	0.176	0.027	[0.129, 0.227]	< 0.001
Meaningfulness × JobStress → life satisfaction	−0.026	0.037	[−0.099, 0.036]	0.473
c’ path (direct effect)				
Perceived union support → life satisfaction	0.022	0.011	[0.008, 0.045]	0.038
Conditional indirect effects				
Lower job stress: perceived union support × JobStress → life satisfaction	0.022	0.004	[0.015, 0.029]	<0.001
Higher job stress: perceived union support × JobStress → life satisfaction	0.018	0.005	[0.009, 0.028]	<0.001
Index of Moderated Mediation (IMM)	−0.003	0.004	[−0.012, 0.004]	0.474

Unstandardized coefficients (b), bias-corrected bootstrap 95% CIs (1,000 samples), SE, standard error. Conditional indirects probe mediation at low (16^th^ percentile) and high (84^th^ percentile) levels of JobStress.

Additional descriptive and subgroup analyses are reported in Appendix Tables A1–A11. The hypothesis tests reported here are central to the theoretical claims.

## 11 Discussion and conclusion

The present study addressed work meaningfulness from a union perspective by examining the roles of perceived union support and its relationship to subsequent wellbeing outcomes. Specifically, we tested two mediation and two moderation hypotheses to investigate whether union support is associated with enhanced work meaningfulness and employee wellbeing, while also exploring how individual difference factors such as status anxiety may influence these relationships. Table 9 reports on hypotheses outcomes.

**Table 9:** A summary of hypotheses outcomes.

H1: Membership → meaningfulness (+)	Regression	Not supported
H2: PUS → meaningfulness (+)	Regression	Supported
H3: PUS → JobSat via meaningfulness	Mediation	Supported
H4: PUS → LifeSat via meaningfulness	Mediation	Supported
H5: Status anxiety moderates the mediation effect of PUS on satisfaction via meaningfulness.	Moderated mediation	Not supported
H6: Job stress moderates the mediation effect of PUS on satisfaction via meaningfulness.	Moderated mediation	Not supported

Our findings showed that perceived union support is positively associated with meaningfulness, which is in turn associated with higher job and life satisfaction. Importantly, this mediated relationship held regardless of employees’ status anxiety and job stress levels. Contrary to Hypotheses 5 and 6, neither status anxiety nor job stress moderated the mediation process. These null findings suggest that the beneficial pathway from union support to well-being through meaningfulness operates similarly across varying psychosocial strain levels. However, the relatively small differences in conditional indirect effects across moderator levels also suggest limited variation in the effect, which may have reduced the likelihood of detecting moderation. Additionally, the limited variability of the job stress measure (SD = 0.64) and its operationalization using a single three-point item may be another reason for the low statistical power for detecting interaction effects. That is why we cautiously claim that employees experiencing high status anxiety or high job stress might still benefit from perceiving their union as supportive, as it helps them derive greater meaning from their work, which might in turn associate with greater satisfaction. The present findings contribute to meaningful-work theory by highlighting the importance of institutional support quality rather than formal membership. From a self-determination perspective (Ryan and Deci, 2017), perceived union support likely contributes to satisfaction of autonomy, competence, and relatedness needs, proximal psychological resources that foster meaningfulness. From the job characteristics tradition (Hackman and Oldham, 1976), unions that deliver concrete improvements to role clarity, task significance, or working conditions may act as job-level resources that increase perceived task significance and impact. Finally, social exchange accounts (Blau, 1964; Eisenberger et al., 2001) suggest that union support produces norms of reciprocity that translate into more positive work attitudes and wellbeing. Together, these perspectives explain why PUS, not mere membership, was consistently associated with greater meaningfulness and why meaningfulness partially transmitted PUS effects to job and life satisfaction.

The study findings refine conceptualizations of meaningful work by foregrounding the quality of institutional support rather than formal organizational membership. Drawing on self-determination theory (Ryan and Deci, 2017), perceived union support likely contributes to the satisfaction of basic psychological needs, autonomy (procedural voice and protection), competence (advocacy for fair treatment and resources), and relatedness (collective solidarity) thereby fostering a stronger sense of meaningfulness in work. This mechanism explains why PUS (not membership) was a relatively more reliable correlate: membership without perceived support provides the institutional label but not the proximal psychological resources that contribute to meaning. From the job-characteristics perspective (Hackman and Oldham, 1976), unions that demonstrably advocate for improved work conditions and clarity of roles can increase task significance and perceived impact, which are core drivers of meaningful work. Thus, perceived union support may operate as an external job-design resource that amplifies the subjective significance of work, consistent with our mediation results linking PUS → meaningfulness → job and life satisfaction. Our results also advance union scholarship by distinguishing instrumental membership from relational support. Prior research on unions and wellbeing has produced mixed findings; our results suggest that the union’s effectiveness in generating member wellbeing depends more on whether members feel supported by the union (Davis, 2012; Blanchflower and Bryson, 2020a; Blanchflower and Bryson, 2020b). Practically, this implies unions’ internal practices (communication, casework, visible wins) matter more for meaning and wellbeing than numerical membership alone. Across models, the *quality* of the union–member relationship (perceived support) rather than membership status accounted for variance in meaningful work and well-being. PUS showed consistent positive associations with meaningfulness, and meaningfulness transmitted these associations to job and life satisfaction (small but reliable indirect effects). In contrast, formal membership was unrelated to meaningfulness or satisfaction. Status anxiety and job stress related to lower meaningfulness, yet they did not alter the benefits of PUS, suggesting the perceived support–meaningfulness link is relatively robust across different psychosocial risk levels. These findings support accounts of meaningful work by highlighting supportive collective institutions as proximal resources for meaning construction at work.

## 12 Implications

Our results extend meaningful-work scholarship in three ways. First, we introduce unions as collective meaning-making agents, broadening a literature that has traditionally emphasized individual-level antecedents such as job design, leadership, or personal calling (Rosso et al., 2010; Martela and Pessi, 2018). By theorizing PUS as a dual construct that combines instrumental resource provision and symbolic solidarity, we expand the conceptual map of meaningfulness at work. Second, by integrating SDT, job characteristics, and social exchange perspectives, we highlight how PUS simultaneously satisfies psychological needs, enriches task significance, and fosters reciprocal attachments. This cross-theoretical integration strengthens understanding of why PUS is more consequential than membership. Third, our null moderation findings challenge deficit-based views of stress and anxiety. Rather than undermining the PUS-meaningfulness relationship, these conditions did not significantly alter it. This might indicate that institutional support functions as a relatively resilient source of meaning, even for workers experiencing psychosocial strain, echoing research on social resources as buffers of vulnerability (Ross and Mirowsky, 2003).

Our study also contributes to debates in industrial relations. In Western contexts, membership itself has been associated with greater satisfaction (Blanchflower and Bryson, 2020a). In Turkey, however, the decoupling of membership and perceived support, driven by political patronage, state-paid dues, and instrumental incentives, might account for why membership does not associate with meaningfulness. In this specific context, union affiliation may be influenced by political alignment, career considerations, or other instrumental motivations, and as such, membership alone may not necessarily translate into positive employee attitudes. Consistent with social exchange perspectives (Rhoades and Eisenberger, 2002) and research on union support (Cardador et al., 2019; Shore et al., 1994; Tetrick et al., 2007), our findings suggest that employees derive greater psychological benefits from feeling supported by their union than from merely holding membership status. Accordingly, unions seeking to strengthen member well-being may benefit from emphasizing responsive communication, accessible representation, professional development opportunities, and visible advocacy regarding members’ workplace concerns. This highlights that the authenticity of support, rather than numerical membership, determines whether unions foster well-being. Our findings therefore complement comparative work showing that union revitalization depends less on density than on building genuine member engagement and trust (Frege and Kelly, 2003; Simms and Holgate, 2010).

For unions, the findings emphasize the importance of shifting from a transactional service model (e.g., benefits administration) toward relational engagement strategies that build solidarity and identity. For organizations, the study suggests that monitoring employee perceptions of meaningfulness should extend beyond internal HR practices to include workers’ experiences with unions and other collective institutions.

Practitioners in trade unions and work organizations could undertake various short-term and longer-term actions to reap the benefits of meaningful work. Trade unions could survey their members for their perceptions of meaningfulness and gauge how they contribute to members’ sense of significance from their work. By understanding their own impact and the alignment between their practices and members’ perceived meaningfulness, they can implement action plans to demand further opportunities or work re-design programs from organizations to increase members’ meaningful experiences.

Our findings suggest that interventions designed to strengthen employees’ perceptions of work meaningfulness may represent a promising avenue for unions and public organizations. Organizations could monitor their employees’ meaningfulness experiences regularly to understand personal and organizational determinants of meaningful work perceptions, try to address aspects of work that hinder or decrease those perceptions, and increase the ones that boost them, and build bridges between employee values and organizational practices to maximize the benefits from meaningful work perceptions. Rather than focusing exclusively on traditional transactional functions such as collective bargaining or material benefits, unions may contribute to employees’ psychological well-being by helping members recognize the broader significance and social value of their work. Examples may include highlighting public service contributions, communicating the societal impact of employees’ efforts, recognizing member achievements, and creating opportunities for participation and voice in workplace decisions.

## 13 Limitations

Several limitations should be acknowledged. Both perceived union support and job stress were measured using single-item indicators. While single-item measures have been used successfully in large-scale survey research and can provide parsimonious assessments of broad perceptions (Wanous et al., 1997), they do not permit reliability estimation and may be more susceptible to measurement error than multi-item scales. This limitation is particularly relevant for perceived union support, which may encompass both instrumental and expressive dimensions that cannot be fully captured by a single rating. Similarly, job stress represents a multifaceted construct involving factors such as workload, role conflict, time pressure, and job insecurity, aspects that were not separately assessed in the present study. Consequently, the findings should be interpreted with appropriate caution.

## Data Availability

The original contributions presented in the study are included in the article/Supplementary material, further inquiries can be directed to the corresponding author.
