# Supplementary material for: Perceived union support, work meaningfulness, and well-being: evidence from an emerging country context
Source: Front Psychol. 2026 Aug 20;17:1839771. doi: 10.3389/fpsyg.2026.1839771 (PMC13537963; doi:10.3389/fpsyg.2026.1839771)
Supplement: Supplementary file 1 [file Data_Sheet_1.PDF]

Appendix

Further analyses with sociodemographic data are presented below.

A1. Public Servants' Union Membership in Turkiye by Confederation

Abbreviation	Confederation	Estimated membership
KESK	Confederation of Public Employees' Trade Unions	166.266
TÜRKİYE KAMU-SEN	Confederation of Turkish Public Workers' Unions	560.060
MEMUR-SEN	Confederation of Public Servants' Trade Unions	1.078.831
BASK	Confederation of Independent Trade Unions of Public Employees	4.524
BİRLEŞİK KAMU-İŞ	Confederation of United Public Employees' Unions	189.332
HAK-SEN	Confederation of Rights of Public Employees' Unions	2.005
ÇALIŞAN SEN	Confederation of Employees' Union Associations	6.139
TÜM MEMUR-SEN	Confederation of All Public Servants' Unions	991
ANADOLU-SEN KONFEDERASYONU	Confederation of Anatolian Public Employees' Unions	784
ŞEHİT GAZİ-SEN KONFEDERASYONU	Confederation of Veterans, Martyrs' Families and Patriotic Public Servants' Unions	1.731
MİL-SEN	Confederation of Moral, Principled and Merit-Based Unions	13.694
YURT-SEN	Confederation of Homeland Unions	1.589
TÜM KAMU-SEN	Confederation of All Public Employees' Unions	484
HÜR SEN	Confederation of Libertarian Unions	31.599
KAMU BİRLİĞİ	Confederation of Public Unity	106.508
BİRLİK SEN	Confederation of Unity of All Public Employees' Unions	3.304
MÜHENDİS TEK-SEN	Confederation of Engineers, Technical Services and Other Public Employees' Unions	4.592
Independent Unions	Independent Unions	146.724
	**TOTAL**	**2.319.157**

A2. Sociodemographic data

Mean age	41.2 (SD=9.11)	Education (0: Below high school 1: High school 2: Vocational school 3: Bachelor's degree 4: Master's or PhD)	0: 8 (0.4%) 1: 83 (5%) 2: 251 (15.0%) 3: 898 (53.7%) 4: 433 (25.9%)
Gender	Woman: 699 (41.8%)	0: No administrative role	0: 446 (26.7%)

(0: woman, 1: man)	Man: 974 (58.2%)	1: Manager/director<br>2: Head of department<br>3: Department chair<br>4: Chief of staff<br>5: Private secretary<br>6: Head of Center/Institute<br>7: General Secretary<br>8: Dean/vice dean<br>9: Other	1: 112 (6.7%)<br>2: 15 (0.9%)<br>3: 25 (1.5%)<br>4: 113 (6.8%)<br>5: 15 (0.9%)<br>6: 5 (0.3%)<br>7: 6 (0.4%)<br>8: 7 (0.4%)<br>9: 929 (%55.5)
Monthly income<br>(0: 30.000-50.000 TL<br>1: 50.000-70.000 TL<br>2: 70.000-90.000 TL<br>3: 90.000-120.000 TL<br>4: 120.000-150.000 TL<br>5: Above 150.000 TL)	0: 410 (24.5%)<br>1: 520 (31.1%)<br>2: 234 (14%)<br>3: 328 (19.6.%)<br>4:118 (7.1%)<br>5: 63 (3.8%)	Kids	0: 520 (31.1%)<br>1: 370 (22.1%)<br>2: 515 (30.8%)<br>3: 210 (12.6%)<br>4: 47 (2.8%)<br>5: 8 (0.5%)<br>6: 1 (0.1%)<br>7: 2 (0.1%)<br>Mean: 1.36 (SD=1.17)
Marital status<br>(0: single, 1:married)	Single: 414 (24.7%)<br>Married: 1259 (75.3%)	Mean years of experience	11 (SD=9.32)
Job and education compatibility<br>(0:yes, 1:no)	Yes: 1179 (70.5%)<br>No: 494 (29.5%)	Labor union membership<br>(0: yes, 1: no)	Yes: 1291(77.2%)<br>No: 382 (22.8%)

A3. Which confederation is your labor union affiliated with?

Which confederation?	Counts	%
MEMUR-SEN	577	44.7 %
OTHER	315	24.4 %
BİRLEŞİK KAMU-İŞ	23	1.8 %
TÜRKİYE KAMU-SEN	247	19.1 %
TÜM MEMUR-SEN	8	0.6 %
HAK-SEN	5	0.4 %
ŞEHİT GAZİ-SEN	5	0.4 %
KESK	33	2.6 %
BAĞIMSIZ	28	2.2 %
MİL-SEN	14	1.1 %

Which confederation?	Counts	%
ANADOLU-SEN	1	0.1 %
TÜM KAMU-SEN	19	1.5 %
HÜR SEN	2	0.2 %
KAMU BİRLİĞİ	5	0.4 %
BİRLİK-SEN	6	0.5 %
ÇALIŞAN-SEN	2	0.2 %
BASK	2	0.2 %

Participants expressed moderate satisfaction with their socioeconomic status (M = 3.08). While 37.6% selected the midpoint of the scale, 27.3% reported being satisfied (rating 4), and 8.9% were completely satisfied (rating 5). In contrast, 26.3% expressed dissatisfaction (ratings 1 or 2). Overall, the distribution suggests a central tendency toward neutral or moderately positive evaluations of socioeconomic standing.

A4. Satisfaction with socioeconomic status

Are you satisfied with your current socioeconomic status? (1: Completely dissatisfied, 5: Completely satisfied)	Counts	%	Mean
1	187	11.2 %	
2	252	15.1 %	
3	629	37.6 %	3.08
4	456	27.3 %	
5	149	8.9 %	

Participants' perceptions of whether their job met expectations were generally favorable (M = 3.43). The largest proportion (42.6%) rated their job as meeting expectations "to a great extent," while 30.7% selected the midpoint, and 10.9% reported complete fulfillment of expectations. Only 15.8% indicated dissatisfaction (ratings 1 or 2), suggesting that most respondents perceived at least moderate congruence between job realities and personal work-life expectations.

A5. Job-life expectations fit

Does your current job meet your expectations from work life? (1: Not at all, 5: Completely)	Counts	%	Mean
1	99	5.9 %	
2	165	9.9 %	
3	513	30.7 %	3.43
4	713	42.6 %	
5	183	10.9 %	

Satisfaction with pay were less favorable. The mean rating was 2.71, below the scale midpoint. Nearly half of participants (44.6%) reported disagreement that their pay reflected their effort

(ratings 1 or 2), while 25.9% agreed (rating 4) and only 4.6% completely agreed (rating 5). About one-quarter (24.9%) chose the neutral midpoint. These findings indicate widespread dissatisfaction with perceived pay adequacy relative to effort. This finding is corraborated by fair pay perceptions data presented right below. Slightly less than half of participants (44%) considered their wages to be comparatively fair, whereas a majority (56%) reported perceiving unfairness. This binary measure reflects a prevalent sense of inequity regarding compensation structures among public sector employees (A6 and A7).

A6. Satisfaction with pay

Do you believe your and others' wage/salary corresponds to your/their effort? (1:Completely disagree, 5: Completely agree)	Counts	%	Mean
1	319	19.1 %	
2	427	25.5 %	
3	416	24.9 %	2.71
4	434	25.9 %	
5	77	4.6 %	

A7. Fair pay perceptions

Do you believe your and others' wage/salary to be comparatively fair? (0: Yes: 1:No)	Counts	%
0	736	44 %
1	937	56 %

A8 illustrates engagement patterns with unions. More than two-thirds of respondents reported never (35.2%) or rarely (33.0%) engaging with their labor union. About one-quarter (25.8%) engaged at a moderate level, while only a small minority reported frequent (3.9%) or consistent (2.0%) involvement. These figures suggest that while union membership is high, active participation remains limited.

A8. How frequently do you engage with your labor union?

The frequency of engagement (1: Never, 5: Always)	Counts	%
Never	455	35.2 %
Rarely	427	33.0 %
Moderately	333	25.8 %
Frequently	51	3.9 %
Always	26	2.0 %

Independent-samples t-tests (A9) revealed significant gender differences across most variables. Women reported higher levels of status anxiety (M = 3.09, SD = 1.06) compared to men (M = 2.68, SD = 1.11), t(1671) = –7.48, p < .001, d = –0.37. Similarly, women experienced greater job stress (M = 2.24, SD = 0.61) than men (M = 2.07, SD = 0.66), t(1671) = –5.19, p < .001, d = –0.25. In contrast, men reported significantly higher perceived work meaningfulness (M = 3.59, SD = 0.53) than women (M = 3.48, SD = 0.48), t(1671) = 4.35, p < .001, d = 0.21. Men also demonstrated higher job satisfaction (M = 3.53, SD = 0.74) than women (M = 3.37, SD = 0.81), t(1671) = 4.02, p < .001, d = 0.19. No significant gender difference emerged for life satisfaction, t(1671) = 0.39, p = .699.

A9. Gender differences across measures

Variables	Women		Men		*t* (1671)	*p*	Cohen's d
	M	*SD*	*M*	*SD*			
Status anxiety	3.09	1.06	2.68	1.11	-7.483	**<.001**	-0.37
Job stress	2.24	0.61	2.07	0.66	-5.186	**<.001**	-0.25
Perceived work meaningfulness	3.48	0.48	3.59	0.53	4.345	**<.001**	0.21
Job satisfaction	3.37	0.81	3.53	0.74	4.015	**<.001**	0.19
Life satisfaction	2.89	0.76	2.90	0.84	0.387	.699	0.01

Note. N = 1673. Woman = 699, Man = 974

One-way ANOVAs demonstrated significant differences in all major outcomes based on whether participants' jobs aligned with their educational background (A10). Participants who reported alignment between their education and job responsibilities experienced lower status anxiety (M = 2.72, SD = 1.09) compared to those without such fit (M = 3.17, SD = 1.09), F(1, 985) = 57.63, p < .001, η² = .03. General stress was also slightly lower in the fit group (M = 2.11, SD = 0.64) relative to the misfit group (M = 2.20, SD = 0.66), F(1, 985) = 6.80, p < .001, η² < .01. Moreover, individuals with job–education fit reported higher work meaningfulness (M = 3.62, SD = 0.48 vs. M = 3.35, SD = 0.53), F(1, 985) = 90.00, p < .001, η² = .05, greater job satisfaction (M = 3.60, SD = 0.74 vs. M = 3.12, SD = 0.83), F(1, 985) = 123.03, p < .001, η² = .07, and higher life satisfaction (M = 2.98, SD = 0.81 vs. M = 2.70, SD = 0.76), F(1, 985) = 44.34, p < .001, η² = .02. Collectively, these results highlight the substantial impact of educational alignment on work-related and general wellbeing.

A10. Differences across job-education fit

Measure	Does your job align with your education?					
	Yes		No		F (1, 985)	η2
	M	*SD*	*M*	*SD*		
Status anxiety	2.72	1.09	3.17	1.09	57.63**	0.03
General stress	2.11	0.64	2.20	0.66	6.80**	0.00
Perceived work meaningfulness	3.62	0.48	3.35	0.53	90.00**	0.05
Job satisfaction	3.60	0.74	3.12	0.83	123.03**	0.07
Life satisfaction	2.98	0.81	2.70	0.76	44.34**	0.02

**p<.001

ANOVA results also revealed systematic differences in psychological outcomes by levels of satisfaction with socioeconomic status (A11). Status anxiety decreased sharply as socioeconomic satisfaction increased, ranging from M = 3.71 (SD = 1.16) among the completely dissatisfied to M = 1.78 (SD = 0.97) among the completely satisfied, $F(1, 546) = 126.5$, $p < .001$, $\eta^2 = .23$. Similarly, general stress declined from M = 2.44 (SD = 0.59) in the lowest satisfaction group to M = 1.77 (SD = 0.66) in the highest group, $F(1, 546) = 30.5$, $p < .001$, $\eta^2 = .06$. Perceived work meaningfulness increased with satisfaction, from M = 3.30 (SD = 0.57) to M = 3.92 (SD = 0.56), $F(1, 546) = 40.2$, $p < .001$, $\eta^2 = .10$. A similar upward trajectory was observed for job satisfaction (M = 3.14, SD = 0.94 vs. M = 4.03, SD = 0.79), $F(1, 546) = 48.8$, $p < .001$, $\eta^2 = .11$, and life satisfaction (M = 2.18, SD = 0.71 vs. M = 3.68, SD = 1.02), $F(1, 546) = 143.3$, $p < .001$, $\eta^2 = .28$. These results suggest that socioeconomic status satisfaction is a powerful correlate of psychological and occupational wellbeing.

A11. Differences across satisfaction with socioeconomic status

Measure	**Are you satisfied with your current socioeconomic status? (1:Completely dissatisfied, 5: Completely satisfied)**										F (1, 546)	η2
	1		2		3		4		5			
	M	*SD*	*M*	*SD*	*M*	*SD*	*M*	*SD*	*M*	*SD*		
Status anxiety	3.71	1.16	3.46	0.90	2.94	0.98	2.40	0.90	1.78	0.97	126.5**	0.23
General stress	2.44	0.59	2.30	0.60	2.15	0.63	2.04	0.63	1.77	0.66	30.5**	0.06
Perceived work meaningfulness	3.30	0.57	3.36	0.50	3.52	0.47	3.65	0.44	3.92	0.56	40.2**	0.10
Job satisfaction	3.14	0.94	3.06	0.86	3.43	0.70	3.67	0.65	4.03	0.79	48.8**	0.11
Life satisfaction	2.18	0.71	2.42	0.61	2.85	0.64	3.26	0.64	3.68	1.02	143.3**	0.28

**p<.001
